# Advocating individual-based profiles of elite athletes to capture the multifactorial nature of elite sports performance

**DOI:** 10.1038/s41598-024-76977-8

**Published:** 2024-11-01

**Authors:** K. Zentgraf, L. Musculus, L. Reichert, L. Will, A. Roffler, S. Hacker, C. Hilpisch, K. Wiedenbrüg, N. Cermak, C. Lenz, H. de Haan, M. Mutz, L. Wiese, A. Al-Ghezi, M. Raab, K. Krüger

**Affiliations:** 1 Goethe University Frankfurt, Institute of Sport Sciences, Ginnheimer Landstrasse 39, Frankfurt am Main, 60487 Germany; 2 German Sport University Cologne, Institute of Psychology, Cologne, Germany; 3 Justus Liebig University Giessen, Institute of Sports Science, Giessen, Germany; 4 Martin Luther University Halle Wittenberg, Institute of Philosophy II, Halle, Germany; 5 Goethe University Frankfurt, Institute of Computer Science, Frankfurt am Main, Germany

**Keywords:** Elite sport, Individual, Expertise, Diagnostics, Physiology, Psychology

## Abstract

Elite athletes are high-performance outliers within their specific sports. Even though science seeks to understand the nature of expertise and elite performance, much knowledge remains compartmentalized within subdisciplines. Despite this multidimensionality being acknowledged, an interdisciplinary approach to understanding elite athletes is still rare. This paper synthesizes insights across scientific domains in order to describe the population and individual characteristics of elite athletes. We analyzed diagnostic data from approximately 300 German squad athletes across eight different sports (e.g., gymnastics, volleyball, ice hockey, 3 × 3 basketball etc., age_female_ = 18.95 ± 4.84 years, age_male_ = 19.32 ± 4.19 years) with expertise values ranging from 2 (*low expertise*) to 16 (*high expertise*). Data covered muscular strength, lower-body dynamics, muscle-power genetics, blood micronutrients, basic cognitive function, mental health, social support, and training conditions. Results of logistic regressions identified basic cognitive function (*B =* 0.89) and well-balanced blood micronutrients (*B =* 1.22) as critical factors distinguishing elite athletes. Additionally, multiple linear regressions suggested that lower-body dynamics (ß* =* 0.72) is related to increasing expertise values. We examined interactions between determinants of elite performance, and found that social support is positively associated with mental health and training conditions, whereas muscular strength correlates with lower-body dynamics. Focusing on top elite athletes in contrast to semi-elite athletes, we found higher within-group similarities in basic cognitive function and blood micronutrients. Findings indicate the need for a systemic, individualized, and comprehensive model using individual-based profiles.

## Introduction

Elite athletes, such as those competing in the Paris 2024 Olympics, are the high-performance outliers within their specific sports. Whereas their sport-specific superior performance is evident from observation alone, what about its not so obvious determinants? Multiple lines of research on sports expertise have focused on high performance as a product of either nature or nurture. For the nurturers, what seems to unite these athletes is extensive experience and practice within their sports^1^. For the ‘naturers’, inherent talent is a prerequisite for elite performance, given only a few attain world and Olympic level. This argument is neatly summarized by the quote: ‘*When all other extrinsic factors (the nurturers) are consistent—the time spent training*,* the type of training*,* the facilities*,* the training environment—what will ultimately distinguish elite performers is their genetic make-up*’^2^. Current discussions clearly indicate a joint nature–nurture explanation. However, quantification is difficult, given that it is generally accepted that physical capability phenotypes are highly polygenic, making it very difficult to quantify the influence of genetic factors on athletic performance^3^.

Recently, there has been a call to move beyond a nature–nurture dichotomy in seeking to find a blueprint for cutting-edge sport performance^4^. But other than accumulating large amounts of deliberate practice or having the respective talent genes, what are these commonalities? Some authors have followed research lines seeming to suggest that ‘challenges’ or even ‘trauma’ paired with early sporting achievement^5–7^ are common precursors for outstanding sporting success. Other authors propose that elite athletes have superior cognitive functioning compared to those who are not engaged in such high levels of physical activity^8^.

We argue that the extensive body of literature remains compartmentalized within subdisciplines, even though research has acknowledged the need to study elite athletes’ brains, and environments in some generalizable and overarching form^9^. A recent review criticized the current state of research for assessing performance indicators and determinants in disciplinary isolation, and it called for a pragmatic turn^10^. Such a pragmatic turn requires researchers to overcome the fact that, for instance, sport psychologists focus on visual search behavior or intrinsic motivation in isolation; motor and exercise scientists focus only on performance assessments related to strength, speed, or movement technique; sport physiologists focus on the capacity of the cardiorespiratory system or recovery^11,12^; and sport sociologists focus solely on elucidating the role of environmental factors such as sport-related social support in their training group or talent-development systems.

Whereas the afore-mentioned empirical research strategies deliver much knowledge, it remains compartmentalized within these subdisciplines. In contrast, in practice, talent decisions, such as the selection of talents, are often based on multifactorial factors. The assessments of coaches, known as the ‘coach’s eye’, are often decisive in this process^13^. These assessments are described as subjective, experience-based, intuitive, and holistic, emphasizing the consideration of many different pieces of information. However, it remains unclear what specific information coaches rely on to make their decisions. Theoretical work, as evident in most modern expertise models, also clearly emphasizes the multifactorial nature of the phenomenon. One recent expertise model by Ullén et al.^14^ shifts attention away from deliberate practice^15^ which has dominated expertise research for quite some time, and opens the field for a multitude of domains that impact on eliteness through their interactions. Recent studies have found that the variance explained by deliberate practice varies, therefore casting doubt on its ultimate impact^16^. For instance, data from Güllich and Barth^17^ stress that not even the presence of extraordinarily high early-career achievements can be validated in the vast majority of future elite athletes. Analyzing individual careers demonstrates high variability in not only their careers but also the determinants of their high performance^18^.

The current study applied Ullén’s gene–environment interaction model, because this aims to conceptualize expertise not as a result of isolated innate factors (‘talent’), but as ‘multifactorial’ and interactive. As a consequence, our empirical research program requires us to assess not only factors related to genetic influence, environmental aspects (‘nurture’), sociopsychological aspects, neural mechanisms, and physical properties, but also their interdependencies. Further, we advocate that beyond needing to assess the multidimensional nature of expertise, we require an individualized approach that looks beyond statistical means and focuses on single athletes’ profiles.

In this paper, we argue, first, that focusing on the individual is crucial, because only a few athletes achieve top performance levels, and their journey to elite status is highly unique and idiosyncratic, second, that it is necessary to include a broad range of determinants of elite performance from different scientific domains to characterize the individual athlete. We hypothesize that outstanding expertise in sports is highly individual and heterogeneous. The current state of the art does not allow us to evaluate heterogeneity within elite sports, because many studies have taken an expert-novice-comparison approach^19,20^. In addition, peak performance is based on extreme selectivity in the sport system: Only one athlete can win the gold medal; only a few athletes achieve Olympic-squad standards in their home country; and the more high-level the squad, the less spots are available. From a scientific perspective, homogeneity is a major issue in elite sports: Applying findings from group-based approaches to a single individual is only feasible when the statistical model holds for each individual. Because this is typically not the case, sport-expertise research might be tagged as being highly nonergodic^21^—in other words, the mean parameter value of a presumably representative sample is not predictive for specific and individual sample members.

Another issue that applies to all expertise research—be it in music, sports, or the arts—is the methodological limitations imposed on the scientific approach: Olympians refrain from participating in experimental research, prospective studies take a long time, and longitudinal studies with elite athletes are laborious and costly. Cross-sectional studies, as a reasonable compromise, fail to capture the dynamic nature of sport careers and athletes’ developmental stages over time. Therefore, the next step to take in expertise research is to acknowledge these multifactorial characteristics and the specific interactions that occur^10^.

This paper aims to synthesize insights across various scientific domains in order to describe population and individual features as well as their associations in current German elite athletes who are preparing for the Olympics. Based on the current literature, we hypothesize that in terms of multidisciplinary performance determinants, elite athletes (classified as repeatedly, internationally top-ranked athletes over several years) are only selectively homogeneous compared to semi-elite athletes. In addition, we expect to find highly individual, nonergodic data patterns of performance determinants in elite athletes. Based on our current data set, models of expertise can then be augmented empirically to disclose their multifactorial nature as well as the ways in which factors interact. In addition, the data will provide practitioners in elite sports with individual, but adequately socially referenced, data on elite athletes that they can use to tailor interventions and counseling.

## Results

We tested the above assumptions by determining whether very successful elite athletes show a homogeneous pattern of ‘superiority’ over eight different multidisciplinary domains. We first used group-based regression statistics to analyze general effects in a homogeneous collective of elite and semi-elite athletes. Then, we analyzed the associations between these eight determinants in correlational statistics to better understand their network structure. Next, we analyzed variances in all eight performance determinants in elite and semi-elite athletes. Finally, we focused on the 22 top elite athletes alone and checked whether their individual profiles could be predicted from the group-based statistics and whether this would be categorized as ergodic.

### Regression approaches

#### Logistic regression analyses

We conducted four logistic regressions to examine the effect of eight predictor variables (scores on muscular strength, lower-body dynamics, muscle-power genetics, blood micronutrients, basic cognitive function, mental health, social support, and training conditions) on the likelihood of the occurrence of one of the following sports expertise levels: (a) ≥ 13, (b) ≥ 13 and controlling for sex, (c) ≥ 13 and controlling for age, and (d) ≥ 13 and controlling for sex and age. We found no multicollinearity for any of the logistic regressions (all variance inflation factors [VIFs] were below 1.5). With regard to the outliers, the various methods showed inconsistent results. Due to these results and the standardized collection of data, we included all cases (*n* = 296) in the analysis.

Model (a) containing all eight predictors was statistically significant, χ²(8) = 24.82, *p* = .002. The model explained 19.6% (Nagelkerke’s *R*²) of the variance in expertise and correctly classified 92.9% of cases.

Model (b), containing all eight predictors and controlling for sex, was also statistically significant, χ²(9) = 25.96, *p* = .002. The model explained 20.4% (Nagelkerke’s *R*²) of the variance in expertise and correctly classified 92.9% of cases.

Model (c), including all eight predictors and controlling for age, was also statistically significant, χ²(9) = 125.06, *p* < .001 and explained 83.8% (Nagelkerke’s *R*²) of the variance in expertise. It correctly classified 97.3% of cases.

Model (d), including only athletes with an expertise level higher than 12 containing all eight predictors plus controlling for age and sex, was also statistically significant, χ²(10) = 126.90, *p* < .001, explaining 84.8% (Nagelkerke’s *R*^2^) of the variance and correctly classifying 97.3% of cases. Detailed results are presented in (Table 1). Descriptive details can be found in Supplementary Table S1.

Table 1 provides the results of all four logistic regressions (a—d) including regression coefficients (*B*), standard errors (*SE*), Wald chi-square statistics (Wald), and 95% confidence intervals (CI) for each predictor.

**Table 1:** Results of the four logistic regressions (**a**) expertise ≥ 13,  (**b**) expertise ≥ 13 and controlling for sex,  (**c**) expertise ≥ 13 and controlling for age, and (d) expertise ≥ 13 and controlling for sex and age.

	*B*	*SE*	Wald	*p*	95% CI
(a) Expertise ≥ 13					
(Constant)	-3.16	0.35	81.87	< 0.001*	
Muscular strength	0.06	0.27	0.05	0.826	[0.55, 1.61]
Lower-body dynamics	-0.12	0.33	0.14	0.706	[0.59, 2.16]
Muscle-power genetics	-0.13	0.26	0.24	0.623	[0.69, 1.88]
Blood micronutrients	1.22	0.50	5.95	0.015*	[0.11, 0.79]
Basic cognitive function	0.89	0.27	10.60	0.001*	[0.24, 0.70]
Mental health	0.28	0.28	0.94	0.332	[0.44, 1.33]
Social support	0.13	0.27	0.24	0.624	[0.52, 1.48]
Training conditions	-0.40	0.26	2.42	0.120	[0.90, 2.47]
(b) Expertise ≥ 13 and controlling for sex					
(Constant)	-3.50	0.50	48.83	< 0.001*	
Muscular strength	0.06	0.27	0.06	0.813	[0.55, 1.60]
Lower-body dynamics	-0.19	0.34	0.31	0.577	[0.64, 2.33]
Muscle-power genetics	0.01	0.29	0.00	0.971	[0.56, 1.74]
Blood micronutrients	1.28	0.51	6.28	0.012*	[0.10, 0.76]
Basic cognitive function	0.89	0.28	10.32	0.001*	[0.24, 0.71]
Mental health	0.35	0.29	1.40	0.237	[0.40, 1.26]
Social support	0.07	0.27	0.07	0.794	[0.55, 1.59]
Training conditions	-0.36	0.26	1.87	0.171	[0.86, 2.37]
*+ Female*	0.61	0.26	1.11	0.292	[0.18, 1.69]
(c) Expertise ≥ 13 and controlling for age					
(Constant)	-27.17	6.99	15.10	< 0.001*	
Muscular strength	-0.18	0.68	0.07	0.789	[0.32, 4.55]
Lower-body dynamics	-0.35	0.73	0.23	0.635	[0.34, 5.92]
Muscle-power genetics	-0.54	0.74	0.53	0.468	[0.40, 7.39]
Blood micronutrients	0.45	0.99	0.20	0.652	[0.09, 4.45]
Basic cognitive function	1.16	0.79	2.14	0.144	[0.07, 1.48]
Mental health	-0.99	0.60	2.71	0.100	[0.83, 8.70]
Social support	0.18	0.45	0.15	0.697	[0.35, 2.04]
Training conditions	0.48	0.58	0.69	0.405	[0.20, 1.92]
*+ Age*	1.00	0.27	14.14	< 0.001*	[0.22, 0.62]
(d) Expertise ≥ 13 and controlling for sex and age					
(Constant)	-29.95	8.40	13.86	< 0.001*	
Muscular strength	-0.12	0.73	0.03	0.865	[0.27, 4.75]
Lower-body dynamics	-0.30	0.76	0.16	0.692	[0.31, 5.99]
Muscle-power genetics	-0.08	0.86	0.01	0.930	[0.20, 5.81]
Blood micronutrients	0.32	1.03	0.10	0.752	[0.10, 5.41]
Basic cognitive function	0.80	0.85	0.88	0.348	[0.08, 2.39]
Mental health	-0.87	0.63	1.93	0.165	[0.70, 8.14]
Social support	-0.07	0.53	0.02	0.888	[0.38, 3.03]
Training conditions	0.69	0.62	1.26	0.263	[0.15, 1.68]
*+ Age*	1.07	0.29	13.28	< 0.001*	[0.19, 0.61]
*+ Female*	2.02	1.58	1.64	0.201	[0.01, 2.92]

*p < .05, elite coded as ‘1’, semi-elite as ‘0’.

#### Multiple linear regression analyses

Prior to performing regression analyses, we tested several assumptions. First, we evaluated linearity by visually inspecting the standardized residuals against the standardized predicted values. A Durbin–Watson test yielded a value of 0.18, falling outside the acceptable range of 1.5 to 2.5 and thereby indicating nonindependent residuals^22^. The Breusch–Pagan test^23^ indicated heteroscedasticity, χ²(8) = 16.03, *p* = .042. VIFs were below the threshold of 10^24^: muscular strength (1.10), lower-body dynamics (1.16), basic cognitive function (1.05), blood micronutrients (1.03), muscle-power genetics (1.09), mental health (1.18), training conditions (1.27), and social support (1.25), indicating no concern for multicollinearity.

The main model (a) including all eight predictors was statistically significant, *F*(8, 287) = 4.36, *p* < .001, and explained 10.8% of the variance in expertise (*R*² = 0.108; adjusted *R*² = 0.084).

Table 2 provides the standardized regression coefficients (β), standard errors (*SE*), *t* values, and significance levels for each predictor.

The regression coefficients indicate that three predictors contribute significantly to the model. Specifically, the first two are the basic cognitive function score (β = 0.71, *p* < .001) and the lower-body dynamics score (β = 0.72, *p* = .006). These two had the strongest impact on expertise, suggesting that a one-unit increase in the basic cognitive function and lower-body dynamics score is associated with an increase in expertise by 0.71 and 0.72 units. Blood micronutrients also had a significant, albeit smaller, effect on expertise (β = 0.53, *p* = .040), with a one-unit increase in blood micronutrients associated with an increase in expertise by 0.53 units.

Taking into consideration that inherently, a higher expertise level (e.g., accumulating international medals and successes) can evolve only with more years in the specific sports and thereby depends on age, and that some of the independent scores are influenced by sex (e.g., muscle-power genetics), we repeated multiple linear regressions, controlling additionally for (b) age, (c) sex, and (d) age and sex. Model (b), controlling for age, was significant, *F*(9, 286) = 47.03, *p* < .001, and explained 59.7% of the variance in expertise (*R*² = 0.597; adjusted *R*² = 0.584). Model (c), controlling for sex, was also significant, *F*(9, 286) = 4.20, *p* < .001, and explained 11.7% of the variance in expertise (*R*² = 0.117; adjusted *R*² = 0.089). Model (d), taking both age and sex into account, still yielded a significant fit, *F*(10, 285) = 45.11, *p* < .001, explaining 61.3% of the variance in expertise (*R*² = 0.613; adjusted *R*² = 0.599). All results are reported in Table 2. Descriptive details can be found in Supplementary Table S2.

Results of the multiple linear regressions including

- all eight predictors.
- all eight predictors and controlling for age.
- all eight predictors and controlling for sex.
- all eight predictors and controlling for age and sex.

**Table 2:** Results of the multiple linear regression including (a) all eight predictors, (b) all eight predictors and controlling for age, (c) all eight predictors and controlling for sex, and (d) all eight predictors and controlling for age and sex.

	β	*SE*	*t*	*p*
(a) All eight predictors				
(Intercept)	6.92	0.18	38.74	< 0.001*
Muscular strength	-0.03	0.19	-0.14	0.885
Lower-body dynamics	0.72	0.26	2.79	0.006*
Muscle-power genetics	-0.08	0.20	-0.40	0.686
Blood micronutrients	0.53	0.26	2.07	0.040*
Basic cognitive function	0.71	0.21	3.39	< 0.001*
Mental health	0.10	0.19	0.52	0.621
Social support	0.11	0.24	0.45	0.652
Training conditions	-0.29	0.22	-1.34	0.182
(b) All eight predictors + age				
(Intercept)	6.95	0.12	58.41	< 0.001*
Muscular strength	-0.11	0.13	-0.81	0.419
Lower-body dynamics	0.49	0.16	2.97	0.003*
Muscle-power genetics	-0.28	0.13	-2.13	0.034*
Blood micronutrients	-0.11	0.18	-0.60	0.548
Basic cognitive function	0.00	0.15	-0.03	0.976
Mental health	-0.11	0.14	-0.77	0.440
Social support	0.18	0.14	1.34	0.182
Training conditions	0.00	0.14	0.03	0.974
*+ Age*	2.43	0.12	19.59	< 0.001*
(c) All eight predictors + sex				
(Intercept)	6.58	0.28	23.29	< 0.001*
Muscular strength	-0.03	0.19	-0.15	0.877
Lower-body dynamics	0.66	0.26	2.53	0.011*
Muscle-power genetics	0.08	0.23	0.34	0.738
Blood micronutrients	0.54	0.26	2.11	0.035*
Basic cognitive function	0.67	0.21	3.19	0.002*
Mental health	0.20	0.21	0.94	0.346
Social support	0.04	0.24	0.16	0.874
Training conditions	-0.27	0.22	-1.24	0.217
*+ Female*	0.70	0.44	1.60	0.111
(d) All eight predictors + age and sex				
(Intercept)	6.50	0.18	35.41	< 0.001*
Muscular strength	-0.11	0.13	-0.85	0.397
Lower-body dynamics	0.41	0.16	2.53	0.012*
Muscle-power genetics	-0.08	0.14	-0.54	0.592
Blood micronutrients	-0.10	0.18	-0.56	0.575
Basic cognitive function	-0.06	0.15	-0.40	0.691
Mental health	0.02	0.14	0.15	0.877
Social support	0.10	0.14	0.70	0.487
Training conditions	0.03	0.13	0.26	0.793
+ *Age*	2.44	0.14	17.96	< 0.001*
*+ Female*	0.90	0.27	3.29	0.001*

*p < .05.

### Association between domains both within and between subdisciplines

To address the interdisciplinary knowledge gap in sports expertise, we examined specific interactions between determinants stemming from different subdisciplines across all athletes. The correlational analysis (*n* = 296; see Supplementary Figure S1 online) considered both within-subdiscipline and between-subdiscipline correlations, highlighting the interconnectedness of these variables and the importance of a holistic perspective on understanding sports expertise. Within each subdiscipline, the analysis revealed several significant correlations. First, there was a significant positive correlation between (maximal) muscular strength and lower-body dynamics (*r* = .282, *p* < .001), indicating a close relationship between overall body strength and lower-body movement efficiency, speed, and power. Second, the correlation between muscle-power genetics and lower-body dynamics was also significant (*r* = .186, *p* = .001), suggesting that genetic predispositions for muscle power are moderately related to enhanced lower-body dynamics.

Within psychosocial variables, there was a significant positive correlation between mental health and social support scores (*r* = .277, *p* < .001)—that is, the higher the perceived sport-related social support, the better athletes’ mental health and vice versa. Furthermore, training conditions also correlated positively with mental health scores (*r* = .305, *p* < .001) and perceived social support (*r* = .386, *p* < .001), suggesting that athletes who perceive more favorable training conditions also perceive better social support.

Interestingly, between-subdiscipline correlations also show interdependencies. Basic cognitive function correlated significantly positively with lower-body dynamics (*r* = .116, *p* = .046), suggesting that the cognitive abilities of athletes might have an impact on speed- and power-oriented physical performance of the legs or vice versa.

Finally, we found a significant negative correlation between muscle-power genetics and social support (*r* = − .138, *p* = .017). We have no theoretical explanation for this association, but assume that it might represent a sample artifact—such as athletes with lower muscle-power genetics randomly more often reporting that they perceive less social support.

### Variance within domains in Elite and Semi-elite athletes

The variance of all predictors was checked for the 22 elite (red-colored bar in each domain predictor circle) as well as 1,000 random samplings of varying 22 semi-elite athletes. The latter are represented by the green-colored boxes. The dashed lines show the range of sampled variances within these semi-elite athletes.

**Fig. 1:**
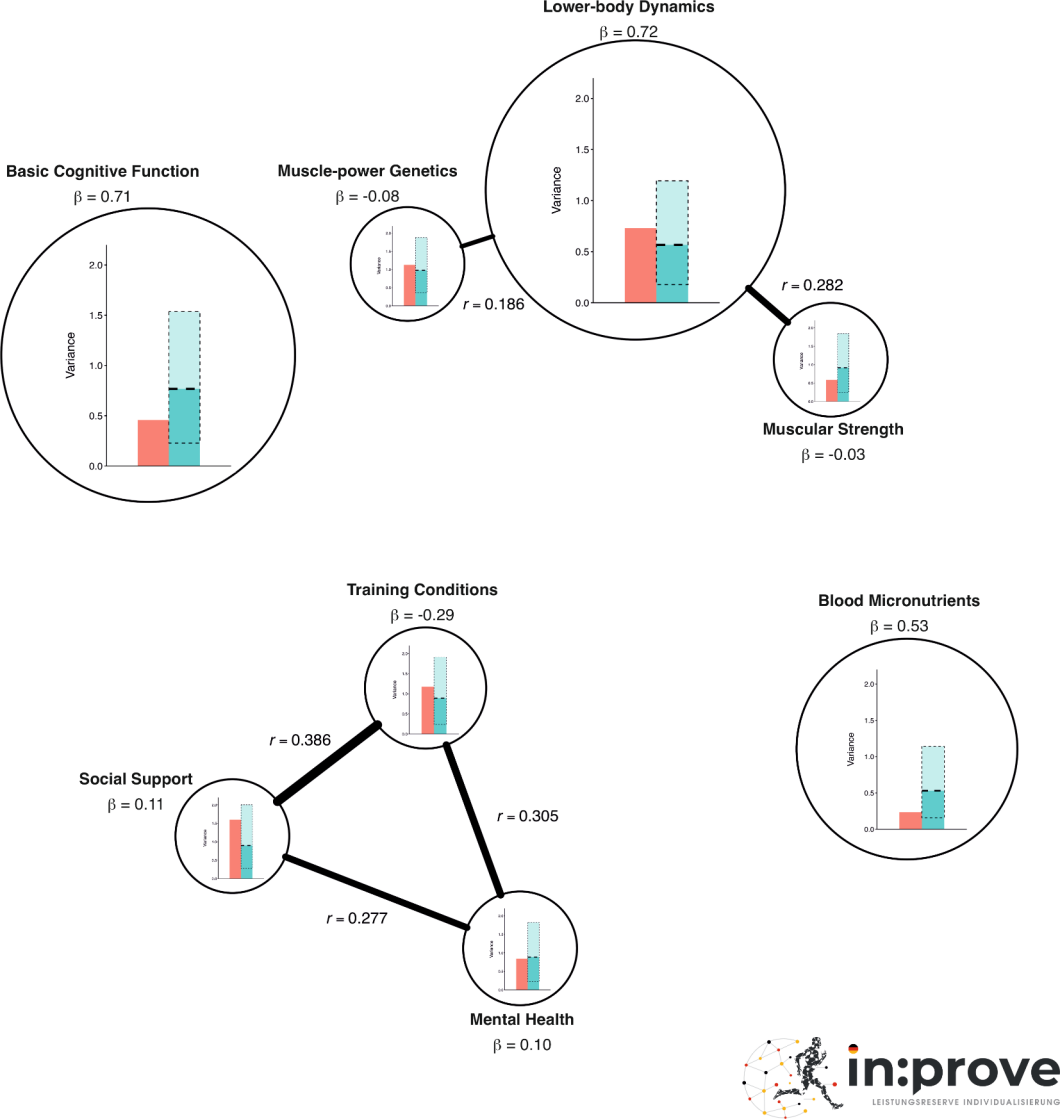
Network plot illustrating the results of all group level statistics. The figure includes (**a**) results of the multiple linear regression of each variable with expertise (size of circle depending on beta coefficient: the larger the circle, the higher the β coefficient), (**b**) results of the correlation analysis (distance between circles according to their correlation: the closer, the higher the correlation; connection lines are displayed only for correlations of *r* > .15: the thicker the line, the higher the correlation), and (**c**) variances of 22 elite and the ranges of sampled (1,000 iterations) variances for 22 semi-elite athletes.

Figure 1 shows that elite athletes exhibit relatively small variance in both their superior basic cognitive functioning and their well-balanced blood micronutrients. However, in perceived social support, the elite athletes even seem to show more variance than semi-elite athletes. In all other predictors, interindividual differences are of a comparable magnitude between all elite and semi-elite athletes indicated by a similar level for the red bar and the dashed line of the green bar.

### Individual profiles

**Fig. 2:**
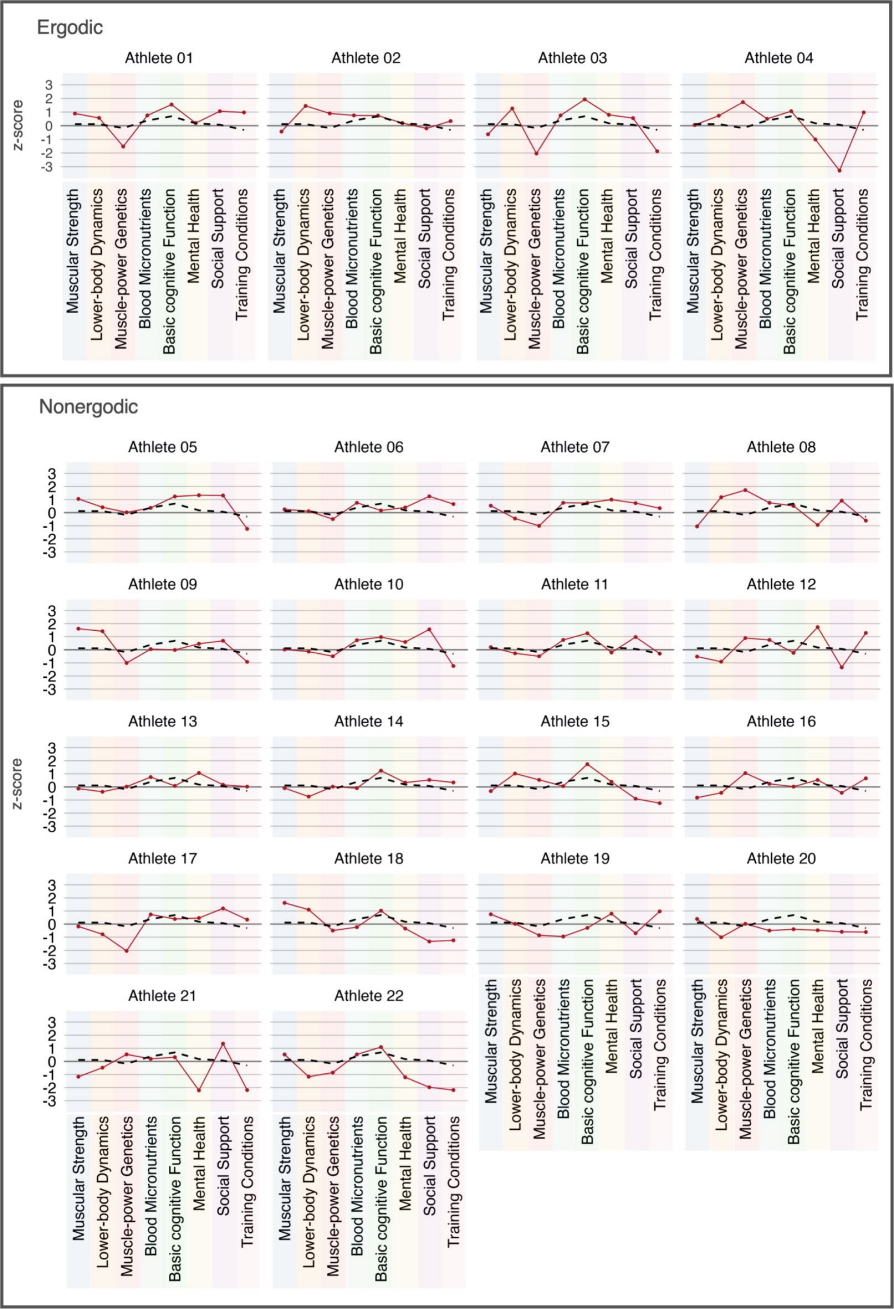
Profile lines showing individual *z* scores for all 22 elite athletes (red line) and the mean *z* score for all 22 elite athletes as reference (dashed black line).

Figure 2 presents the individual profiles of the 22 elite athletes alone (expertise values ≥ 13). These profiles illustrate domain-specific athletes’ superiorities and inferiorities based on z scores. Of these 22 elite athletes, 19 have at least one domain with an exceptionally positive ‘outlier’ (*z* scores of > 1.0). On the other hand, the majority (15 of 22 athletes) also show at least one domain with a particularly low value (*z* scores of < -1.0). Considering the z scores as indicating socially referenced relative superiority and inferiority, 18 out of 22 elite athletes are characterized by a positive sum score (all individual *z* scores from eight domains). Checking for ergodicity based on the group-level results in (a) superior basic cognitive functioning, 12 elite athletes have higher *z* scores than the mean of the 22 elite athletes and 15 elite athletes have higher *z* scores than 296 semi-elite athletes. In (b) lower-body dynamics, only 9 elite athletes have higher *z* scores than the mean of the 22 elite athletes and 8 elite athletes have higher *z* scores than 296 semi-elite athletes. In (c) blood micronutrients, 13 elite athletes have higher *z* scores than the mean of the 22 elite athletes and 15 elite athletes have higher *z* scores than 296 semi-elite athletes. However, a combination of higher *z* scores in superior basic cognitive functioning, lower-body dynamics, and blood micronutrients—as might be derived from the group-based statistics—is displayed individually in only four elite athletes: Athletes 01, 02, 03, and 04. These athletes could cautiously be considered ergodic, whereas the rest of the elite athletes could be considered as nonergodic (Fig. 2). When checking for superior values related to the results of the logistic regression (i.e., superiority only in basic cognitive functioning and blood micronutrients), three more athletes (10, 11, 22) show similarity to the group-based statistics.

A value below zero therefore means the athlete showed an inferior value in this variable than the average of 296 athletes. A value above zero represents a superiority compared to the sample of 296 athletes. Based on group-level results, athletes were categorized into ergodic and nonergodic. Four athletes showed a superior value in all basic cognitive function, lower-body dynamics, and blood micronutrients (ergodic). All other athletes did not show superior performance on these variables (nonergodic).

## Discussion

This study aimed to use a multifactorial and multidomain expertise model to analyze German national squad athletes. It sought to challenge the idea that long-standing and very successful elite athletes would show a homogeneous pattern of ‘superiority’ over eight different multidisciplinary domains. To assess this, we first used group-based statistics to analyze general effects in a homogeneous collective of elite and semi-elite athletes. Second, we analyzed the associations between these eight determinants to better understand their network structure. Third, to look for similarities within groups of athletes, we analyzed variances in all eight performance-predicting determinants in elite and semi-elite athletes. Fourth, we focused on the 22 top elite athletes alone and checked whether their individual profiles could be predicted from the group-based statistics and could be categorized as ergodic.

The regression analyses with eight predictors were performed on a logistic (binary code of elite and semi-elite) as well as on a (continuously scaled) multivariate basis. These data suggest that only two predictors have a consistent impact on sport expertise: These are basic cognitive functioning and blood micronutrients. Additionally, in the multiple-regression model, lower-body dynamics relates to expertise.

To evaluate the relevance of these predictors, it has to be considered that the regression models controlling for age strongly lower the beta coefficients for the predictors. This is because higher age is a prerequisite for persistent international sport-related success, and only a minor number of athletes earn Olympic medals. The regression models without these control variables yield beta coefficients ranging from 0.53 to 0.73 for single significant predictors. This suggests that with an increasing expertise level, the higher the probability that elite athletes will have improved basic cognitive functioning, exhibit fewer insufficiencies in blood micronutrients, and have superior lower-body dynamics.

In addition, our data suggest that the variance is lower among elite athletes (see Fig. 1) than semi-elite athletes—that is, elite athletes have similar characteristics in terms of basic cognitive functioning and blood micronutrients. In the other predictors, in contrast, the magnitudes of variance are comparable to those of semi-elite athletes. In perceived social support, the 22 German elite athletes are very dissimilar (i.e., high variance) and seem to exhibit even more differences than the semi-elites (Fig. 1, red- and green-colored bars in the social support circle).

Looking at the internal structure of all eight predictors, constructs are associated more closely when they are in one disciplinary domain than when they are between domains. Motor-performance parameters such as muscular strength, muscle-power genetics, and lower-body dynamics share some common variance. As hypothesized on the basis of earlier studies, the explained variance from muscle-power genetics to lower-body dynamics is about 10%^25^. This, however, also means that 90% of the variance has to be explained via other factors than genetics such as optimal and consistent power-oriented practice and training, high standards in technique and skill, and cognitive skills for maintaining practice or for tapping the full potential of power tasks. Other studies suggest that genetics explain more variance in athletic performance^26^. This is due primarily to the varying genetic influence on specific components of performance such as response to training stimuli, resilience to injuries and illnesses, and nutrient absorption. These can differ in line with the unique demands of each sport or discipline. Furthermore, the polygenic nature of many of these factors complicates any precise definition of the genomic profile of elite athletes^27^.

In addition, the social-science-oriented determinants suggest some closer associations: Perceived training conditions, mental health, and social support share around 10 to 20% of variance. This is in line with other studies focusing on psychosocial environmental factors in German elite sports^28^, but also those on elite sports in other countries^29^. Two performance determinants show low associations with other variables. These are basic cognitive functioning and blood micronutrients. Both seem unrelated to the other determinants. A new finding is that basic cognitive functioning and blood micronutrients are the factors with a significant impact in the group-based statistics. It might be inferred that elite athletes stand out by employing superior information processing (i.e., mental speed, as well as selective attention) measured with a validated concentration and attention test. This is also in line with current meta-analyses^8,30^, and it might be speculated that this is pronounced in sports disciplines that require speedy processing of visual stimuli in interactive settings such as 3 × 3 basketball, volleyball, or ice hockey. Therefore, it has to be noted that this result is influenced by the selection of the sports disciplines and might differ in, for instance, endurance athletes.

Regarding blood micronutrients, research has demonstrated that an adequate supply is essential for enhancing athletic performance and supporting training adaptations^31^. To ensure an adequate level of nutrient intake, regular blood tests are conducted, a balanced diet is maintained, and micronutrient supplements are used as needed to address any deficiencies^32^. However, within the context of athletic expertise, it is important to note that micronutrient levels can be more variable over time compared to other performance factors, and they are subject to short-term fluctuations^33^.

The present study is cross-sectional, and the dynamics of these characteristics will be analyzed more closely when the ongoing project called in:prove allows longitudinal analyses. Additionally, it must be noted that age and training age are preconditions to reach high expertise values, making this an ecologically valid but methodologically problematic assessment of expertise. However, to refrain from categorizing elite athletes or tagging every athlete simply as elite or novice has both been massively disputed as misleading in expertise research^34^.

When expertise research aims to analyze general features of internationally successful athletes, it often makes an assumption about homogeneity. We object to this general fiction that there is one factor explaining eliteness. Instead, we assume that the road to excellence is unique, idiosyncratic and individual. Indeed, the individual profiles of 22 German elite athletes fail to consistently achieve above-average scores in all eight domains. In the same vein, they even exhibit inferior values in some domains. Individual profiles show, however, no consistent pattern. They illustrate that the highest levels of sport expertise can be attained via a multitude of distinct characteristics and combinations thereof. It is also evident that there were no domains in which an above-average score is a—homogeneously—necessary condition for achieving the highest level of expertise. Nevertheless, these elite athletes possess a greater number of more pronounced superiorities than inferiorities overall, and these might enable an adequate compensation for individual inferiorities. Athlete 20, however, made it to the top although she or he had only one clearly positive *z* score. Athletes 01, 02, 03, and 04 (i.e., 4 out of the 22 athletes) actually show a result pattern that represents the average statistical property of all athletes. Therefore, sport expertise research is nonergodic. Nonetheless, researchers often seek to find the general secret code of eliteness based on statistics that are inadequate when sample sizes are low. In fact, our data suggest that elite athletes are characterized by a very individual pattern of eight features, each of which has been commonly associated with sport expertise in isolation. It might also be speculated that a mixed (i.e., negative and positively balanced) pattern is necessary to compensate for potential ‘inferiorities.’ A comparable proposition was advanced in balance theory^35^, which was initially developed to describe human performance in work environments. The tenets of balance theory posit that the coexistence of both positive and negative elements within a complex and dynamic work environment is an inherent and unavoidable phenomenon. However, it is possible to strengthen the positive elements to counterbalance the negative ones. For optimal performance, it is not a single feature that is of paramount importance, but rather that the overall equation is such that the facilitating aspects outweigh the negative ones that impede performance. From a domain-encompassing and multifactorial perspective on elite sports, a high level of basic cognitive functioning, perceived social support, or well-balanced blood micronutrients might counteract low muscle-power genetics or little social support. And this pattern of compensation might be highly individual. With this current cross-sectional data set, we additionally cannot capture the temporal dynamics in each athlete over their career. But what our findings imply is that there is no single blueprint for sport eliteness, and that above-average cognitive functioning and an adequate access to micronutrients seem to be potent facilitators in being an elite athlete. We recommend that future research should focus on individual profiles in order to capture the temporal and structural details of achieving and maintaining an elite status and to evaluate the (im)balance between these factors in elite athletes.

## Methods

### Subjects

The analyses in this study were based on a dataset gathered from 296 professional athletes (age_female_ = 18.95 ± 4.84 years, age_male_ = 19.32 ± 4.19 years; 3 × 3 basketball *n* = 20 female, *n* = 15 male; ice hockey *n* = 20 female, *n* = 34 male; volleyball *n* = 33 female, *n* = 65 male; artistic gymnastics *n* = 17 female; trampoline gymnastics *n* = 13 female, *n* = 13 male; rhythmic gymnastics *n* = 25 female; table tennis *n* = 6 female, *n* = 8 male; modern pentathlon *n* = 13 female, *n* = 14 male). All athletes belonged to a German junior (*n* = 91 female, *n* = 106 male) or senior national team (*n* = 43 female, *n* = 56 male). Members of a junior national team were typically under the age of 20 years (age_female_ = 16.55 ± 1.70 years, age_male_ = 17.27 ± 1.56 years) and they had usually just started competing at international level. Members of a senior national team (age_female_ = 22.86 ± 5.69 years, age_male_ = 24.38 ± 4.36 years) had been competing at the highest level for several years and generally belonged to the highest German national squads (such as prospective or Olympic squads). Some of these athletes have qualified for the 2024 Olympic Games, won medals or prepare for the next events. According to our classification of expertise (see section Expertise level), 22 athletes were classified as elite whereas 274 were classified as semi-elite. Prior to testing, all athletes (additionally the parents for minors) received detailed written and verbal information regarding diagnostics, and they provided written informed consent in accordance with the Declaration of Helsinki. The study protocol was approved by the Ethics Committee of the Justus Liebig University Giessen (ethical approval number: AZ 55/22; approval date: 10 May 2022).

### Design and data acquisition

The present study was conducted with a cross-sectional dataset acquired between February 2022 and December 2023. This was used to synthesize insights across various scientific domains in order to describe both the population and the individual characteristics of current and future elite athletes. Data acquisition took place at junior or senior national team training camps for which national athletes had been nominated. At the beginning of each athlete’s assessment, they carried out the *Zahlenverbindungstest* (see basic cognitive function below) as a group-based diagnostic test. Subsequently, athletes warmed up individually (running, mobility, dynamic stabilization, and coordination tasks) and completed all further diagnostic tests in a permuted order as described below.

### Diagnostic tests

#### Muscular strength

As a proxy measure for overall muscular strength^36^, grip strength was measured unimanually using a hand-held dynamometer (MicroFET2, Hoggan Scientific, Salt Lake City, USA) according to the procedure described in Reichert et al.^37^. Absolute grip strength [N] was assessed and relative strength [N/kg] was calculated by dividing the absolute strength by the athlete’s body weight. Relative grip strength was *z* standardized according to the athlete’s sex, national team, and sport discipline. Subsequently, all *z* scores were summarized to form the variable muscular strength.

#### Lower-body dynamics

Lower-body dynamics consists of two motor performance variables that reflect discipline-specific speed- and power-related components of the lower extremities. The included speed-related components are either tapping performance (©Voss, Doberschütz, Germany) or a 10 m sprint (©Microgate, Bolzano, Italy). During tapping tests in sports, athletes have to generate as many alternating foot contacts as possible on a contact mat over a five-second period, from which the maximum tapping frequency [Hz] was taken. The 10 m sprint performance was measured in seconds [s]. The power-related component consists of jumping performance in either the countermovement jump, drop jump (Microgate, Bolzano, Italy), or sergeant jump (Jump-and-Reach lab-built device). For the countermovement and sergeant jump performance, jump height [cm] was measured, while the drop jump performance was quantified by assessing the Reactive Strength Index (jump height/contact time). For further processing, the best trial from the respective diagnostics was used.

Subsequently, the sport-specific requirements were considered when computing lower-body dynamics. Accordingly, lower-body dynamics consist of tapping and countermovement jump performance in basketball, gymnastics, trampoline, and volleyball; tapping and drop jump performance in rhythmic gymnastics and table tennis; 10 m sprint and countermovement jump performance in ice hockey; and tapping and sergeant jump performance in modern pentathlon. Each variable was *z* standardized separately according to the athlete’s sex and national team and then summarized as lower-body dynamics.

#### Muscle-power genetics

DNA was extracted from human whole blood samples using the Chemagic Magnetic Separation Module I (Perkin Elmer Chemagen Technology Inc., Baesweiler, Germany). Genotyping was performed using the Illumina Global Screening Array + Medical Disease + Psych content (GSAv3.0 + MD + Psych; Illumina Inc, San Diego, CA, USA). Muscle-power genetics refers to an athlete’s genetic predisposition for muscular power performance and consists of a sex-specific polygenic score generated in accordance with Reichert et al.’s findings^25^. In male athletes, single nucleotide polymorphisms of the genes *AGT* (angiotensinogen, rs699), *IP6K3* (inositol hexakisphosphate kinase 3, rs6942022), and *VDR* (vitamin D receptor, rs1544410) were included in the polygenic score; in female athletes, polymorphisms of the genes *ACTN3* (actinin alpha 3, rs1815739), *AGT* (angiotensinogen, rs699), *HSD17B14* (hydroxysteroid 17-beta dehydrogenase 14, rs7247312), *MTRR* (5-methyltetrahydrofolate-homocysteine methyltransferase reductase, rs1801394), and *UCP2* (uncoupling protein 2, rs660339). Polygenic scores were *z* standardized over all athletes and then summarized as muscle-power genetics.

#### Blood micronutrients

A total of 25 milliliters of peripheral venous blood was drawn, and the serum was centrifuged. From the serum, the concentrations of vitamin B12 [pg/ml], 25-OH-vitamin D [ng/ml], folic acid [ng/ml], and ferritin [ng/ml] were analyzed using a chemiluminescent immunoassay. An overall score for blood micronutrients was computed in three steps: First, supply for each micronutrient was coded according to medical norms as either substandard (0; vitamin B12 < 211 pg/ml; 25-OH-vitamin D < 20 ng/ml; folic acid < 3.15 ng/ml), suboptimal (1; vitamin B12 = 211 to 350 pg/ml; 25-OH-vitamin D = 20 to 30 ng/ml; folic acid = 3.15 to 6.8 ng/ml), or within the standard (2; vitamin B12 ≥ 350 pg/ml; 25-OH-vitamin D ≥ 30 ng/ml; folic acid ≥ 6.8 ng/ml). Ferritin was coded sex- and age dependently as either substandard (0; male under 18 years < 14 ng/ml; male over 18 years < 20 ng/ml; female under 18 years < 13 ng/ml; female over 18 years < 10 ng/ml) or within the standard (2; male under 18 years ≥ 14 ng/ml; male over 18 years ≥ 20 ng/ml; female under 18 years ≥ 13 ng/ml; female over 18 years ≥ 10 ng/ml). Second, the scores of available supply conditions were summed up. Third, this sum was divided by twice the number of available scores and then multiplied by 100. Thus, the score for blood micronutrients ranges from 0 to 100 with a score of 100 corresponding to an optimal supply of all available micronutrients. These overall scores were *z* standardized over all athletes and then summarized as blood micronutrients.

#### Basic cognitive function

Participants’ general cognitive performance was assessed using two tasks designed to evaluate lower cognitive functions. The paper-and-pencil-version of the *Zahlenverbindungstest* (German equivalent to the Trail-Making Task Part A;^38^) measures information processing ability (i.e., mental speed). Participants first completed two practice trials, connecting numbers from 1 to 20 as quickly as possible, without worrying about performing neat strokes. They then completed four test pages, connecting numbers from 0 to 100 in ascending order within 30 s per page. The dependent variable was the average number of connected numbers over the four pages, with higher scores indicating faster processing speed. Selective attention was measured using the electronic d2-R test^39^, a validated concentration and attention test. Participants worked through rows of letters, crossing out the letter ‘d’ with two dashes while ignoring other stimuli. After five practice trials, they completed 14 screen pages, each with 60 objects, within 20 s per page. The higher the concentration score, calculated by subtracting wrongly marked objects from correctly marked targets, the better participants’ selective attention. Each variable was *z* standardized separately and then summarized as basic cognitive function.

#### Psychosocial and environmental factors

An online questionnaire was used to assess both psychosocial indicators and factors related to the sporting environment under the assumption that these are potentially associated with elite performance. Detailed information on the structure and thematic content of the questionnaire can be found in the publication by Hilpisch et al.^28^. For the purposes of this study, we were primarily interested in three areas, namely (a) mental health, (b) social support, and (c) training conditions. Regarding the athletes’ *mental health*, we used the German version of the Patient Health Questionnaire-4 (PHQ-4^40^), to ask how often participants had experienced symptoms of anxiety and depression in the past two weeks. The 2-item anxiety scale and the 2-item depression scale can each be answered on a 4-point Likert scale ranging from 1 (*never*), to 4 (*almost every day*). In addition, and because elite athletes have to deal with numerous sport-related stressors, we used the Perceived Stress Scale 4 (PSS-4^41^), to assess respondents’ subjective experience of stress over the previous four weeks. For each of the four statements, respondents could choose one of five possible answers ranging from 1 (*almost never*) to 5 (*almost always*). To obtain an overall score, we first calculated the mean value of the corresponding items for both the PHQ-4 and the PSS-4. Both scales were thus transformed into an identical range and then combined into one mean score. Finally, this total score was *z* standardized as mental health.

Furthermore, athletes’ perceived *social support* can be considered a motivator and driving factor for participation and retention in the elite sport system. Therefore, we used the Perceived Available Support in Sport Questionnaire (PASS-Q^42^). This scale consists of 16 items covering four different dimensions—namely, emotional, esteem, informational, and practical support—without asking about the source of this support. For each statement, athletes were asked to choose one of five response options ranging from 1 (*strongly disagree*) to 5 (*strongly agree*). To gain a more complete picture of athletes’ social support, we also asked about their perceived support from people outside of sport using the Multidimensional Scale of Perceived Social Support (MSPSS^43^). This scale contains 12 statements about the level of social support received from three sources: family, friends, and significant others. Respondents indicated their agreement on a 5-point Likert scale ranging from 1 (*strongly disagree*) to 5 (*strongly agree*). To summarize the athletes’ general social support in a single score, we first calculated the means of the respective items for the PASS-Q and for the MSPSS, before transforming both scales to the same range and then averaging them. This score was then *z* standardized as social support.

Moreover, the online questionnaire also contained items assessing the perceived quality of *training conditions*. We asked athletes to indicate their satisfaction with (a) their training conditions and (b) their coaching staff. Respondents answered both items on a 10-point Likert scale ranging from 1 (*not at all satisfied*) to 10 (*completely satisfied*). After calculating the mean value of both items, the total score was *z* standardized as training conditions.

#### Expertise level

The expertise level was conceptualized on the basis of Swann et al.’s taxonomy for sport expertise^34^. This evaluates athletes in terms of their performance level, success, and experience in their sports. In particular, it codes the highest level they have competed at (category A, scale 1–4), their success at the highest level of competition (category B, scale 0–4), the number of years of experience at the highest level (category C, scale 1–4), and the number of years with international discipline-specific top (ten) rankings at senior level (category D, scale 0–4).

In detail, for the level of competition (category A), the scale 1–4 corresponds to the following performance levels: 1 (regional/lower national level), 2 (national/lower international level), 3 (international level), and 4 (highest ranked international tournaments). It is important to note that this is coded in a discipline- or sport-specific manner. Details of the coding scheme can be found as Supplementary Table S3. For success at the highest level of competition (category B), a scale 0–4 corresponds to the following categories: 0 (participation in the national [German, GER] championship), 1 (1–3 rank in the national [GER] championship or participation in continental championship [e.g., European championship]), 2 (1–3 rank in a continental championship or participation in a world championship), 3 (1–3 rank in the world or participation at the Olympics), and 4 (1–3 rank at the Olympics). For years of experience at the highest level (category C), the scale 1–4 corresponds to the following time periods: 1 (0–2 years), 2 (3–4 years), 3 (5–8 years), and 4 (8–12 years). For years of competition at the international level as a senior athlete (category D), the scale 0–4 corresponds to the following time periods: 0 (no experience), 1 (1–3 years), 2 (3–7 years), 3 (7–10 years), and 4 (> 10 years).

For each athlete, the individual record was obtained from official websites and coded according to the discipline- or sport-specific coding scheme (see Supplementary Table S3). In addition, for the binomial logistic regression, athletes with an expertise level ≥ 13 were classified as elite and those with an expertise level ≤ 12 as semi-elite.

#### Data management

In the in:prove project, we have designed a structured, sustainable, and secure database to host the specific in:prove data. To manage the database, we have developed multiple modules of functional systems that aim to facilitate the secure collection, importing, and exporting of data. Moreover, we have added functional systems to share the data and the results of our analyses with the users of the system in their multiple roles. Our server’s architecture is composed of four working nodes in one cluster. This distributed architecture ensures the required system security, reliability, and performance.

Due to the private nature of the data, we have applied a secure scheme with multiple levels of encryption. We have also applied pseudonymization techniques, with the most sensitive parts of the data being stored separately in a dedicated working node with a more secure environment. These are mainly those parts of the data that could identify users and are not required for the usual data analysis processes. The system technology stack includes React and React Native for the frontend, Node.js for the backend, as well as PostgreSQL and MongoDB for the database management systems.

### Statistical analysis

Data are presented as means ± standard deviations. Statistical analyses were conducted using IBM SPSS Statistics 29.0.2.0 (©IBM Corp., Armonk, New York, USA), as well as R version 4.4.0 (R Core Team, R Foundation for Statistical Computing, Vienna, Austria, URL https://cran.r-project.org/), and RStudio version 2024.4.2.764 (Posit team, Posit Software, PBC, Boston, MA, USA, URL https://posit.co/download/rstudio-desktop/). The significance level for analyses was set to α = 0.05. For all analyses, the expertise level was used as dependent variable and the following eight variables from the different subdisciplines served as predictor variables: muscular strength, lower-body dynamics, muscle-power genetics, blood micronutrients, basic cognitive function, mental health, social support, and training conditions.

All figures were also created within RStudio version 2024.4.2.764 using the igraph package^44^ for Fig. 1 and the ggplot2 package^45^ for Fig. 2.

#### Logistic regressions

The binomial logistic regression analyses were carried out using IBM SPSS Statistics 29.0.2.0 (IBM Corp., Armonk, New York, USA). Based on their expertise level, athletes were binary coded into elite (expertise value ≥ 13) and semi-elite (expertise value ≤ 12). These groups were then used as the dependent variable in all binomial logistic regression analyses. Linearity was tested using the Box–Tidwell method^46^. VIFs were used to test for collinearity. A VIF value of more than 10 indicates the presence of multicollinearity. Outliers were determined using several methods including Studentized residuals (higher or lower than 3;^47^), leverage values (greater than 0.2;^48^), and Cook’s distance (Cook’s *D* < 1;^49^).

#### Multiple regressions

Multiple linear regression analyses were performed using R version 4.4.0 (R Core Team, R Foundation for Statistical Computing, Vienna, Austria) and RStudio version 2024.4.2.764 (Posit team, Posit Software, PBC, Boston, MA, USA). We used visual inspection of scatterplots of the standardized residuals versus the standardized predicted values to assess linearity. A Durbin–Watson test was conducted to check for independence of errors. Visual inspection and a Breusch–Pagan test were implemented to analyze the assumption of homoscedasticity. Given a violation of the homoscedasticity assumption, we calculated heteroscedasticity-consistent (HC) standard errors of type HC3. If additionally, nonnormal errors were present, we used HC4 in accordance with Hayes and Cai^50^. We calculated HC3 and HC4 with the R package ‘lmtest’ version 0.9–40^51^. We analyzed multicollinearity and influential factors using VIFs and Cook’s distance calculated with the R package ‘car’ version 3.1-2^52^.

#### Associations within and between subdisciplines

To analyze specific interactions between the determinants stemming from different subdisciplines across all athletes, we conducted correlational analyses as an exploratory tool. Because of the prior *z* transformation, we calculated one-sided Pearson correlations.

#### Variance between domains in elite and semi-elite athletes

Interindividual variances were calculated separately for elite and semi-elite athletes. To compare variances between both groups according to their sample size, variances for semi-elites were calculated in an iterative process using R version 4.4.0 (R Core Team, R Foundation for Statistical Computing, Vienna, Austria) and RStudio version 2024.4.2.764 (Posit team, Posit Software, PBC, Boston, MA, USA). More specifically, the iterative process (iterations = 1,000) consisted of sampling 22 random athletes out of the semi-elite group and calculating the mean variance for each variable in the respective iteration. Subsequently, mean as well as minimal and maximal variance were calculated over all iterations. Variances of elite and the range of sampled variances of semi-elite athletes were then plotted separately for each variable. In addition, the igraph package^44^ was used to create a network plot containing the results of the multiple linear regression with expertise and the correlation analysis between all eight variables. The size of the circles corresponds to the β coefficients: the larger the circle, the higher the β coefficient. The distance between the circles corresponds to their correlation (the closer, the higher the correlation). Connecting lines are displayed only for correlations *r* > .15 (the thicker the line, the higher the correlation). Afterwards, to create Fig. 1, we included variance plots in the network plot using CorelDraw Graphics Suite 2021 for Mac (Version 23.1.0.389, Corel Corporation, Canada, URL https://www.coreldraw.com/de/pages/download/).

#### Individual profiles

Individual profiles were visualized for the 22 elite athletes to gain more specific information about an athlete’s score distribution. Each *z* score of an individual elite athlete was displayed in a graph. Because the mean of the *z* scores over the whole sample of 296 athletes equals approximately zero, the profile lines stand in relation to the baseline equaling zero, and they represent positive or negative differences to the mean of the whole sample of 296 athletes. A value above zero therefore means that an athlete has a higher value in this specific domain than the average of the athlete population of all 296 athletes. A negative value means that an athlete has a lower score than the average of the athlete population of all 296 athletes. The mean values for each score of the elite athlete sample were also calculated and visualized as a reference (see Fig. 2). Profiles were categorized based on ergodicity to the group-level statistics of the multiple regression analyses.

## Electronic supplementary material

Below is the link to the electronic supplementary material.

Supplementary Material 1

## Data Availability

Data cannot be made fully available publicly because this could lead to elite athletes being identified. However, a subportion of the data as z score means with standard deviations that is deidentified can be obtained on demand from the corresponding author.
