# Supplementary material for: Advocating individual-based profiles of elite athletes to capture the multifactorial nature of elite sports performance
Source: Sci Rep. 2024 Nov 1;14:26351. doi: 10.1038/s41598-024-76977-8 (PMC11530532; doi:10.1038/s41598-024-76977-8)
Supplement: Supplementary file 1 — Supplementary Material 1 [file 41598_2024_76977_MOESM1_ESM.pdf]

Title

Advocating individual-based profiles of elite athletes to capture the multifactorial nature of elite sports performance

Author list

Zentgraf, K.*, Musculus, L., Reichert, L., Will, L., Roffler, A., Hacker, S., Hilpisch, C., Wiedenbrüg, K.,

Cermak, N., Lenz, C., de Haan, H., Mutz, M., Wiese, L., Al-Ghezi, A., Raab, M., and Krüger, K.

***corresponding author**

Supplementary Figure S1.

Correlation matrix of eight performance determinants.

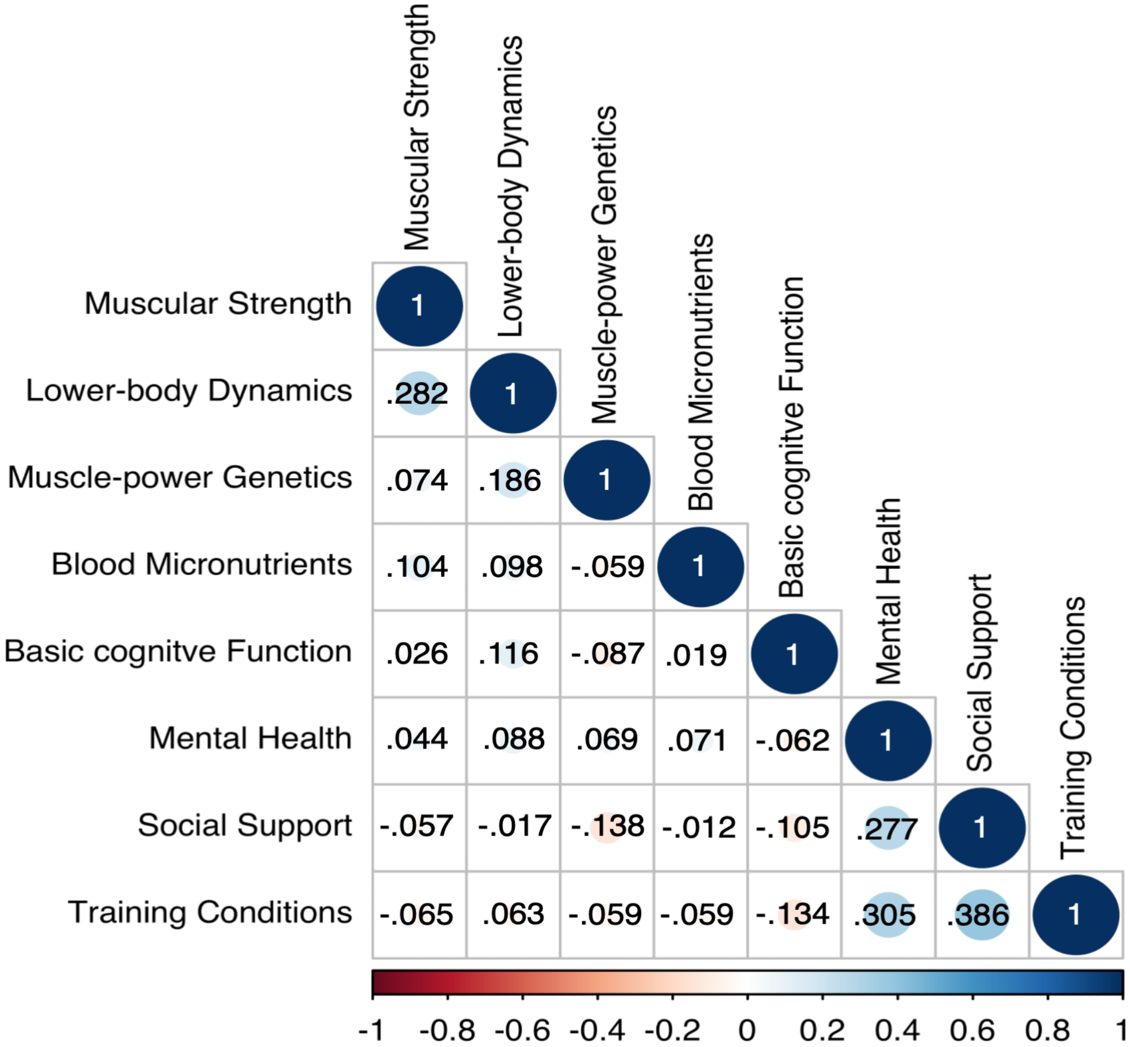

Supplementary Table S1.

Descriptive table with the results of all eight domains separated by expertise level (1 = Elite, 0 = Semi-Elite).

		Valid	Missing	*M*	*SD*	Min.	Max.
Expertise	1	22	0	14.32	1.09	13.00	16.00
Expertise	0	274	0	6.39	2.45	2.00	12.00
Muscular strength	1	22	0	0.12	0.77	-1.17	1.62
Muscular strength	0	274	0	-0.01	0.96	-2.37	2.53
Lower-body dynamics	1	22	0	0.11	0.86	-1.17	1.45
Lower-body dynamics	0	274	0	0.04	0.76	-2.36	2.22
Muscle-power genetics	1	22	0	-0.18	1.06	-2.04	1.72
Muscle-power genetics	0	274	0	0.01	0.99	-2.56	2.60
Blood micronutrients	1	22	0	0.38	0.49	-0.95	0.75
Blood micronutrients	0	274	0	-0.02	0.73	-2.56	0.75
Basic cognitive function	1	22	0	0.69	0.68	-0.40	1.93
Basic cognitive function	0	274	0	-0.02	0.88	-2.12	2.50
Mental health	1	22	0	0.18	0.92	-2.22	1.73
Mental health	0	274	0	0.03	0.95	-3.02	1.73
Social support	1	22	0	0.07	1.27	-3.29	1.56
Social support	0	274	0	0.05	0.96	-3.04	1.56
Training conditions	1	22	0	-0.31	1.08	-2.19	1.29
Training conditions	0	274	0	0.08	0.95	-3.14	1.29

Supplementary Table S2.

Descriptive table with the results of all eight domains.

**Variable of interest**	***M***	***SD***	**Min.**	**Max.**
Expertise	6.98	3.16	2.00	16.00
Muscular strength	5.07×10-4	0.95	-2.37	2.53
Lower-body dynamics	0.04	0.76	-2.36	2.22
Muscle-power genetics	-5.86×10-4	0.99	-2.56	2.60
Blood micronutrients	0.01	0.72	-2.56	0.75
Basic cognitive function	0.03	0.89	-2.12	2.50
Mental health	0.04	0.94	-3.02	1.73
Social support	0.05	0.98	-3.20	1.56
Training conditions	0.05	0.97	-3.14	1.29

Supplementary Table S3.

Coding Scheme 'Expertise' (adapted to Swann et al., 2015).

		A = Athletes highest level of competition [scale: 1-4]				B = Success at the athletes' highest level of competition (e.g., best placement) [scale: 0-4]					C = Number of years of experience at the highest level [scale: 1-4]				D = Number of years with international discipline-specific top (ten) rankings at senior level [scale: 0-4]					Expertise
	in:prove Definition	1 - Regional/lower national level	2 - National/lower international level	3 - International level	4 - Highest ranked international tournaments	0 - Participation at the national (GER) championship	1 - Placement 1-3 at the national (GER) championship or participation at continental championships (e.g., European championship)	2 - Placement 1-3 European championship or participation World championship (or comparable competitions)	3 - Placement 1-3 World championship or participation Olympic Games (or comparable competitions)	4 - Placement 1-3 Olympic Games	1	2	3	4	0	1	2	3	4	= A + B + C + D
Volleyball	Junior	National league / cup	National team matches (e.g., WEVZA)	Youth European Championship	Youth World Championship / Youth Olympic Games	National league participation	(1-3) national league or cup / participation Youth European Championship	(1-3) Youth European Championship / participation Youth World Championship	(1-3) Youth World Championship / participation Youth Olympic Games	(1-3) Youth Olympic Games	0-2 years	3-4 years	5-8 years	8-12 years	No top (ten) ranking at senior level yet	1-3 years	3-7 years	7-10 years	> 10 years	
	Senior	National league / cup	CEV Cup / Challenge Cup	European Championship / Nations League / Champions League	World Championship / Olympic Games	National league participation	(1-3) national league or cup / participation Youth European Championship or Nations League	(1-3) European Championship, Nations League or Champions League / participation World championship or FIVB World Championship	(1-3) World Championship or FIVB World Championship / participation Olympic Games	(1-3) Olympic Games	0-2 years	3-4 years	5-8 years	8-12 years	No top (ten) ranking at senior level yet	1-3 years	3-7 years	7-10 years	> 10 years	
Ice hockey	Junior	National league	Playoffs	National team matches (e.g., comparison of countries)	Youth World Championship / Youth Olympic Games	National league participation	(1-3) national league / participation Youth European Championship	(1-3) Youth European Championship / participation Youth World Championship	(1-3) Youth World Championship / participation Youth Olympic Games	(1-3) Youth Olympic Games	0-2 years	3-4 years	5-8 years	8-12 years	No top (ten) ranking at senior level yet	1-3 years	3-7 years	7-10 years	> 10 years	
	Senior	National league	Playoffs	Continental Cup / Champions Hockey League	World Championship / Olympic Games	National league participation	(1-3) national league / participation European Championship	(1-3) European Championship / participation World Championship	(1-3) World Championship / participation Olympic Games	(1-3) Olympic Games	0-2 years	3-4 years	5-8 years	8-12 years	No top (ten) ranking at senior level yet	1-3 years	3-7 years	7-10 years	> 10 years	
3x3 basketball	Junior	Regional / national tournaments	"Lower" International Tournaments / Series (European Cup / Series)	"Higher" International Tournaments / Series (World Cup / World Series)	Youth World Championship / Youth Olympic Games	National league participation	(1-3) national league / participation Youth European Championship	(1-3) Youth European Championship / participation Youth World Championship	(1-3) Youth World Championship / participation Youth Olympic Games	(1-3) Youth Olympic Games	0-2 years	3-4 years	5-8 years	8-12 years	No top (ten) ranking at senior level yet	1-3 years	3-7 years	7-10 years	> 10 years	
	Senior	Regional / national tournaments	"Lower" International Tournaments / Series (European Cup / Series)	"Higher" International Tournaments / Series (World Cup / World Series)	World Championship / Olympic Games	National league participation	(1-3) national league / participation European Championship	(1-3) European Championship / participation World Championship	(1-3) World Championship / participation Olympic Games	(1-3) Olympic Games	0-2 years	3-4 years	5-8 years	8-12 years	No top (ten) ranking at senior level yet	1-3 years	3-7 years	7-10 years	> 10 years	
Rhythmic gymnastics	Junior	Regional qualification for national championship	National (GER) championship / international tournaments by invitation	Youth European Championship	Youth World Championship / Youth Olympic Games	National league participation	(1-3) national league / participation Youth European Championship	(1-3) Youth European Championship / participation Youth World Championship	(1-3) Youth World Championship / participation Youth Olympic Games	(1-3) Youth Olympic Games	0-2 years	3-4 years	5-8 years	8-12 years	No top (ten) ranking at senior level yet	1-3 years	3-7 years	7-10 years	> 10 years	
	Senior	Regional qualification for national championship	National (GER) championship / international tournaments by invitation	European Championship / World Games / European Games / World Cups / World Challenge Cups	World Championship / Olympic Games	National league participation	(1-3) national league / participation European Championship	(1-3) European Championship / participation World Championship	(1-3) World Championship / participation Olympic Games	(1-3) Olympic Games	0-2 years	3-4 years	5-8 years	8-12 years	No top (ten) ranking at senior level yet	1-3 years	3-7 years	7-10 years	> 10 years	
Trampoline	Junior	Regional competitions / cups	National league / national cups	Youth European Championship	Youth World Championship / Youth Olympic Games	National league participation	(1-3) national league / participation Youth European Championship	(1-3) Youth European Championship / participation Youth World Championship	(1-3) Youth World Championship / participation Youth Olympic Games	(1-3) Youth Olympic Games	0-2 years	3-4 years	5-8 years	8-12 years	No top (ten) ranking at senior level yet	1-3 years	3-7 years	7-10 years	> 10 years	
	Senior	Regional competitions / cups	National league / national cups	European Championship	World Championship / Olympic Games	National league participation	(1-3) national league / participation European Championship	(1-3) European Championship / participation World Championship	(1-3) World Championship / participation Olympic Games	(1-3) Olympic Games	0-2 years	3-4 years	5-8 years	8-12 years	No top (ten) ranking at senior level yet	1-3 years	3-7 years	7-10 years	> 10 years	
Artistic gymnastics	Junior	National league	Youth national cup / Youth international cups	Youth European Championship	Youth World Championship / Youth Olympic Games	National league participation	(1-3) national league / participation Youth European Championship	(1-3) Youth European Championship / participation Youth World Championship	(1-3) Youth World Championship / participation Youth Olympic Games	(1-3) Youth Olympic Games	0-2 years	3-4 years	5-8 years	8-12 years	No top (ten) ranking at senior level yet	1-3 years	3-7 years	7-10 years	> 10 years	
	Senior	National league	National cups / lower international cups	European Championship (World Games / European Games / World Cup / World Challenge Cups)	World Championship / Olympic Games	National league participation	(1-3) national league / participation European Championship	(1-3) European Championship / participation World Championship	(1-3) World Championship / participation Olympic Games	(1-3) Olympic Games	0-2 years	3-4 years	5-8 years	8-12 years	No top (ten) ranking at senior level yet	1-3 years	3-7 years	7-10 years	> 10 years	
Table tennis	Junior	Regional / national tournaments	National championship / national cups and ranking list	Youth European Championship / World Cup / World Tour	Youth World Championship / Youth Olympic Games	National league participation	(1-3) national league / participation Youth European Championship	(1-3) Youth European Championship / participation Youth World Championship	(1-3) Youth World Championship / participation Youth Olympic Games	(1-3) Youth Olympic Games	0-2 years	3-4 years	5-8 years	8-12 years	No top (ten) ranking at senior level yet	1-3 years	3-7 years	7-10 years	> 10 years	
	Senior	Regional / national tournaments	National championship / national cups and ranking list	European Championship / World Cup / World Tour	World Championship / Olympic Games	National league participation	(1-3) national league / participation European Championship	(1-3) European Championship / participation World Championship	(1-3) World Championship / participation Olympic Games	(1-3) Olympic Games	0-2 years	3-4 years	5-8 years	8-12 years	No top (ten) ranking at senior level yet	1-3 years	3-7 years	7-10 years	> 10 years	
Modern pentathlon	Junior	National competitions	National championship / national ranking list	Youth European Championship / World Cup / World Tour	Youth World Championship / Youth Olympic Games	National league participation	(1-3) national league / participation Youth European Championship	(1-3) Youth European Championship / participation Youth World Championship	(1-3) Youth World Championship / participation Youth Olympic Games	(1-3) Youth Olympic Games	0-2 years	3-4 years	5-8 years	8-12 years	No top (ten) ranking at senior level yet	1-3 years	3-7 years	7-10 years	> 10 years	
	Senior	National competitions	National championship / national ranking list	European Championship / World Cup / World Tour	World Championship / Olympic Games	National league participation	(1-3) national league / participation European Championship	(1-3) European Championship / participation World Championship	(1-3) World Championship / participation Olympic Games	(1-3) Olympic Games	0-2 years	3-4 years	5-8 years	8-12 years	No top (ten) ranking at senior level yet	1-3 years	3-7 years	7-10 years	> 10 years	
